# *In Vitro* Exposure of Primary Human T Cells and Monocytes to Polyclonal Stimuli Reveals a Basal Susceptibility to Display an Impaired Cellular Immune Response and Develop Severe COVID-19

**DOI:** 10.3389/fimmu.2022.897995

**Published:** 2022-07-01

**Authors:** Rebeca Viurcos-Sanabria, Aarón N. Manjarrez-Reyna, Helena Solleiro-Villavicencio, Salma A. Rizo-Téllez, Lucía A. Méndez-García, Victoria Viurcos-Sanabria, Jacquelina González-Sanabria, América Arroyo-Valerio, José D. Carrillo-Ruíz, Antonio González-Chávez, Jose I. León-Pedroza, Raúl Flores-Mejía, Octavio Rodríguez-Cortés, Galileo Escobedo

**Affiliations:** ^1^ Laboratory of Immunometabolism, Research Division, General Hospital of Mexico “Dr. Eduardo Liceaga”, Mexico City, Mexico; ^2^ PECEM, Facultad de Medicina, Universidad Nacional Autónoma de México, Mexico City, Mexico; ^3^ Posgrado de Ciencias Genómicas, Universidad Autónoma de la Ciudad de México, Mexico City, Mexico; ^4^ Research Directorate, General Hospital of Mexico “Dr. Eduardo Liceaga”, Mexico City, Mexico; ^5^ Unit for Stereotactic and Functional Neurosurgery, General Hospital of Mexico “Dr. Eduardo Liceaga”, Mexico City, Mexico; ^6^ Faculty of Health Sciences, Anahuac University, Mexico City, Mexico; ^7^ Clínica de Atención Integral para Pacientes con Diabetes y Obesidad, General Hospital of Mexico “Dr. Eduardo Liceaga”, Mexico City, Mexico; ^8^ Departament of Intensive Medical Therapy, General Hospital of Mexico “Dr. Eduardo Liceaga”, Mexico City, Mexico; ^9^ Facultad de Medicina, Universidad Nacional Autónoma de México, Mexico City, Mexico; ^10^ Laboratorio 103, Sección de Estudios de Posgrado e Investigación, Escuela Superior de Medicina, Instituto Politécnico Nacional, Plan de San Luis y Díaz Mirón, Casco de Santo Tomas, Ciudad de México, Mexico

**Keywords:** T cell, monocyte, SARS-CoV-2, COVID-19, PD-1, IL-2, IFN-gamma, IFN-alpha

## Abstract

The contribution of the cellular immune response to the severity of coronavirus disease 2019 (COVID-19) is still uncertain because most evidence comes from patients receiving multiple drugs able to change immune function. Herein, we conducted a prospective cohort study and obtained blood samples from 128 unvaccinated healthy volunteers to examine the *in vitro* response pattern of CD4+ and CD8+ T cells and monocyte subsets to polyclonal stimuli, including anti-CD3, anti-CD28, poly I:C, severe acute respiratory syndrome coronavirus type 2 (SARS-CoV-2) recombinant spike S1 protein, and lipopolysaccharide. Then, we started a six-month follow-up and registered 12 participants who got SARS-CoV-2 infection, from whom we retrospectively analyzed the basal immune response pattern of T cells and monocytes. Of the 12 participants infected, six participants developed mild COVID-19 with self-limiting symptoms such as fever, headache, and anosmia. Conversely, six other participants developed severe COVID-19 with pneumonia, respiratory distress, and hypoxia. Two severe COVID-19 cases required invasive mechanical ventilation. There were no differences between mild and severe cases for demographic, clinical, and biochemical baseline characteristics. In response to polyclonal stimuli, basal production of interleukin-2 (IL-2) and interferon (IFN-) gamma significantly decreased, and the programmed cell death protein 1 (PD-1) increased in CD4+ and CD8+ T cells from participants who posteriorly developed severe COVID-19 compared to mild cases. Likewise, CD14++CD16- classical and CD14+CD16+ non-classical monocytes lost their ability to produce IFN-alpha in response to polyclonal stimuli in participants who developed severe COVID-19 compared to mild cases. Of note, neither the total immunoglobulin G serum titers against the virus nor their neutralizing ability differed between mild and severe cases after a month of clinical recovery. In conclusion, using *in vitro* polyclonal stimuli, we found a basal immune response pattern associated with a predisposition to developing severe COVID-19, where high PD-1 expression and low IL-2 and IFN-gamma production in CD4+ and CD8+ T cells, and poor IFN-alpha expression in classical and non-classical monocytes are linked to disease worsening. Since antibody titers did not differ between mild and severe cases, these findings suggest cellular immunity may play a more crucial role than humoral immunity in preventing COVID-19 progression.

## Introduction

The severe acute respiratory syndrome coronavirus type 2 (SARS-CoV-2) is the causal agent of coronavirus disease 2019 (COVID-19) (1). The clinical presentation of COVID-19 has caught the attention of the scientific community around the globe because of its enormous heterogeneity, ranging from mild and moderate self-limiting viral infection to severe and critical illness (2). However, the mechanisms involved in COVID-19 progression and severity are still a matter of debate (3). A growing body of evidence has pointed out advanced age, male gender, and comorbidities such as hypertension and type 2 diabetes (D) as leading risk factors for developing severe COVID-19 (4–7). Nevertheless, several studies have consistently reported that mild COVID-19 can present even in advanced-age men with comorbidities (2, 6), bringing to light the need to understand additional factors explaining how severe disease develops.

Numerous studies have informed that an exacerbated inflammatory response worsens the clinical course of SARS-CoV-2 infection by increasing the local and systemic levels of tumor necrosis factor-alpha (TNF-alpha), interleukin (IL-) 1 beta, and IL-6 (7–9). However, emerging evidence suggests that a defective cellular immunity may also accelerate COVID-19 progression (10). Compared to patients developing mild symptoms, CD4+ and CD8+ T cell populations from COVID-19 patients admitted to intensive care units (ICU) show reduced production of IL-2, a cytokine with major functions in enhancing T and B cell proliferation (11, 12). Furthermore, CD4+ T cells also express interferon-gamma (IFN-gamma), a cytokine able to inhibit viral replication; however, seriously ill COVID-19 patients show reduced IFN-gamma-producing CD4+ T cells compared to convalescent individuals (10). CD4+ and CD8+ T cell populations from COVID-19 patients in need of hospitalization also show increased expression of cell exhaustion markers, including the programmed cell death protein 1 (PD-1) (13–15). In parallel, the ability of classical and non-classical monocytes to release the antiviral cytokine interferon-alpha (IFN-alpha) decreases as COVID-19 severity increases in patients critically ill compared to those developing mild-to-moderate disease (16). Of note, even though T cells and monocytes appear to lose the capacity of producing key antiviral cytokines, patients with severe COVID-19 exhibit similar neutralizing antibody titers to those found in subjects with mild symptoms (10). This whole evidence emphasizes the idea that impairment in cellular immunity may play a crucial role in worsening SARS-CoV-2 infection.

Although this information suggests that cellular immunity plays a role in COVID-19 progression by stimulating T cells and monocytes to release crucial antiviral cytokines, most of this evidence comes from cross-sectional clinical studies with polytreated patients, making it challenging to correct data interpretation. We hypothesize that cytokine production mediated by cells such as T lymphocytes and monocytes against the SARS-CoV-2 has basal response patterns with the ability to predispose a patient towards the development of either mild or severe disease. However, this question is hard to respond to in infected patients receiving multiple drug schemes and therapeutic maneuvers able to modify the immune response pattern during the COVID-19 course.

For this reason, we conducted a prospective, longitudinal follow-up for six months in 105 family members of medical staff caring for COVID-19 patients, exploring their immune response pattern to *in vitro* polyclonal stimuli simulating the exposure to SARS-CoV-2. After grading disease severity in the participants resulting infected during that period, we retrospectively examined how CD4+ and CD8+ T cells and monocyte subpopulations responded *in vitro* to anti-CD3, anti-CD28, poly I:C, spike protein, and lipopolysaccharide (LPS) in seeking a basal immune pattern that could associate with the development of mild or severe COVID-19.

## Materials and Methods

### Participants and Ethical Disclosures

We invited 128 healthy adult women and men to participate in the study. We enrolled participants if they met the following inclusion criteria: family members of health care professionals working at a dedicated COVID-19 hospital in Mexico City, aging 18-65 years old, negative testing for SARS-CoV-2 by quantitative-polymerase chain reaction (qPCR), and seronegative to anti-SARS-CoV-2 IgG antibodies. We excluded subjects from the study if they had the previous diagnosis of the human immunodeficiency virus (HIV), hepatitis C virus (HCV), hepatitis B virus (HBV), chronic kidney or liver disease, cancer, autoimmune diseases, endocrine disorders, and infectious diseases. We also excluded pregnant or lactating women and patients taking immunomodulatory medication for the last six months. We eliminated participants from the study if they received any vaccine against COVID-19 within six months of enrollment. All study participants provided written informed consent previously approved by the institutional ethical committee of the General Hospital of Mexico (registration number of the ethical code approval: DI/20/501/03/17). The study rigorously met the principles described in the 1964 Declaration of Helsinki and its posterior amendment in 2013.

### Study Design

This prospective, longitudinal study with retrospective data analysis took place from December 2020 to September 2021. All family members of health care professionals who agreed to take part in the study signed the informed consent and received a full explanation of the purposes and procedures of the study. We collected demographic, clinical, and biochemical data from all 128 participants at the enrollment. Demographic and clinical data included sex, age, and previous diagnosis of obesity (body mass index (BMI) > 30 kg/m2), type 2 diabetes (D), hypertension, coronary heart disease (CHD), and hypercholesterolemia. Biochemical data included serum albumin, total proteins, blood glucose, lipid profile, liver and kidney function tests, hematic biometry, C-reactive protein (CRP), and lactate dehydrogenase (LDH). We measured all laboratory parameters using the Beckman Coulter DxC 700 AU Chemistry Analyzer (Beckman Coulter Inc., Brea, CA, USA), the Coulter LH 780 Hematology Analyzer (Beckman Coulter Inc., Brea, CA, USA), and the BCS^®^ XP System (Siemens Healthcare GmbH, Erlangen, Germany). We collected 6 ml venous blood samples from all 128 healthy participants at the enrollment, using tubes containing sodium heparin (VacutainerTM, BD Diagnostics, NJ, USA). After whole blood *in vitro* exposure to polyclonal stimuli, we performed flow cytometry staining for cell surface and intracellular markers of T cells and monocytes, storing flow cytometry data. Polyclonal stimuli were used to simulate the exposure to SARS-CoV-2 in *in vitro* culture settings, using the SARS-CoV-2 recombinant spike S1 protein (the main surface antigen of the virus), Poly I:C (a double-stranded RNA widely used to mimic viral infections *in vitro*), anti-human CD3 and anti-human CD28 (co-stimulatory signals that enhance T cell expansion and activation *in vitro*), and LPS (an endotoxin that enhances monocyte activation). We weekly followed-up to all participants by phone calls for six months, asking for the occurrence of symptoms such as headache, fever (body temperature > 37.5°C), dry cough, tiredness, myalgia, arthralgia, nasal congestion, runny nose, anosmia, dysgeusia, sore throat, diarrhea, shortness of breath, chest pain, and blue-colored skin or lips. After reporting at least one of the above symptoms, participants attended the General Hospital of Mexico for SARS-CoV-2 infection confirmation by qPCR in nasopharyngeal swabs. Then, we started a daily follow-up on each participant confirmed for SARS-CoV-2 infection, recording relevant clinical and biochemical data at the symptom onset and seven days after, and categorizing the development of COVID-19 as mild-to-moderate or severe-to-critical disease. We classified the level of COVID-19 severity according to the World Health Organization (WHO) criteria as follows: mild COVID-19 cases showed headache, fever, dry cough, tiredness, myalgia, arthralgia, nasal congestion, runny nose, anosmia, dysgeusia, sore throat, and/or diarrhea that participants handled at home without needing of oxygen supply or hospitalization; severe COVID-19 cases presented at least one of the above symptoms plus oxygen saturation level (SpO_2_) ˂ 92% on room air, respiratory distress ˃ 30 breath per minute, and/or ˃50% lung involvement on imaging that required either hospitalization or mechanical ventilation. Once we confirmed the clinical outcome of COVID-19, we retrospectively analyzed flow cytometry data for mild or severe groups in seeking a basal immune response pattern to *in vitro* polyclonal stimuli that could associate with the disease severity.

### Cell Cultures

We collected 6 ml venous blood samples from all participants at the enrollment, using tubes containing sodium heparin (VacutainerTM, BD Diagnostics, NJ, USA). Immediately after, we divided each whole blood sample into 24-well ultra-low attachment surface cell-culture plates (Costar, Kennebunk, ME, USA), adding 200 μl blood plus 400 μl RPMI-1640 (Sigma Aldrich, St. Louis, MO, USA) supplemented with 5% fetal bovine serum (FBS), 2 mM L-glutamine, and 10 nM HEPES buffer (GibcoTM, Grand Island, NY, USA) per well in triplicate. We designated the first three wells as unstimulated T cell culture control containing 200 μl blood plus 400 μl supplemented RPMI-1640 for 2 hours. The subsequent three wells had 200 μl blood plus 400 μl supplemented RPMI-1640 incubated in the presence of 0.5 μg/ml SARS-CoV-2 recombinant spike S1 protein (Arigo Biolaboratories, Hsinchu City, Taiwan), 100 μg/ml Poly I:C (Sigma Aldrich, St. Louis, MO, USA), and 10 ng/ml anti-human CD3 and anti-human CD28 (BioLegend, Inc., San Diego, CA, USA) for 2 hours. We designated the following three wells as unstimulated monocyte culture control containing 600 μl blood plus 1.2 ml supplemented RPMI-1640 for 2 hours. The last three wells had 600 μl blood plus 1.2 ml supplemented RPMI-1640 incubated in the presence of 0.5 μg/ml SARS-CoV-2 recombinant spike S1 protein (Arigo Biolaboratories, Hsinchu City, Taiwan), 100 μg/ml Poly I:C (Sigma Aldrich, St. Louis, MO, USA), and 10 ng/ml LPS (Sigma Aldrich, St. Louis, MO, USA) for 2 hours. We incubated cell cultures at 37°C in humidified 5% CO_2_ atmosphere for 2 hours. We selected a 2-hour stimulation based on time-response curves (**Supplementary Figure 1**). We treated whole blood cultures for intracellular staining with 1:1000 Brefeldin A (BioLegend, Inc., San Diego, CA, USA) and 1 μg/ml monensin (BioLegend, Inc., San Diego, CA, USA) for 45 min before the culture’s ending.

### Immunostaining and Flow Cytometry

After incubation, we collected whole blood samples into 5 ml falcon tubes (BD, Bedford, MA, USA), centrifuged tubes at 500 g for 10 min, and washed cell pellets with 200 μl PBS 1X (Sigma Aldrich, St. Louis, MO, USA) twice. Immediately after, we added 5 ml ammonium-chloride-potassium (ACK) lysing buffer to each cell pellet, mixed gently and incubated for 10 min at room temperature. After centrifuging each tube at 500 g for 10 min, we discarded the supernatant and resuspended the white blood cell (WBC) pellet in 1 ml PBS 1X (Sigma Aldrich, St. Louis, MO, USA). After an extra washing step, we resuspended 5x10^5^ WBCs in 50 μl cell staining buffer (BioLegend, Inc., San Diego, CA, USA). For monocyte cultures, we incubated WBCs with 5 μl True-Stain Monocyte Blocker™ (BioLegend, Inc., San Diego, CA, USA) for 10 min on ice. Then, we added anti-CD14 APC Fire 750, anti-CD16 PE/Cy5, and anti-HLA-DR PE (BD Biosciences, San Jose, CA, USA) for 20 min in darkness at 4°C. For T cell cultures, we incubated WBCs with anti-CD3 APC Fire 750, anti-CD4 BV 510, anti-CD8 APC, and anti-PD-1 PE for 20 min in darkness at 4°C. Afterward, we incubated WBCs with 100 μl Fixation Medium A (FIX & PERMTM Cell Permeabilization Kit) (Invitrogen™, Carlsbad, CA, USA) for 20 min at room temperature. After rinsing WBCs with Cell Staining Buffer (BioLegend, Inc., San Diego, CA, USA), we incubated cells with 100 μl Permeabilization Medium B (FIX & PERM TM Cell Permeabilization Kit, Invitrogen™, Carlsbad, CA, USA) plus anti-IFN-gamma Pacific Blue and anti-IL-2 PE/Cy7 for T cell cultures or IFN-alpha AF 647 (BioLegend, Inc., San Diego, CA, USA) for monocyte cultures during 20 min in darkness at room temperature. After rinsing the cell pellets with cell staining buffer (BioLegend, Inc., San Diego, CA, USA), we acquired cells on a BD FACS Canto II Flow Cytometer (BD Biosciences, San Jose, CA, USA), acquiring 10,000 events per test on CD3+ or HLA-DR+ cells, respectively in three different and individual staining.

### Gating Strategy

After we confirmed the severity of COVID-19, we retrospectively analyzed flow cytometry data for mild COVID-19 cases or severe COVID-19 participants. For T cells, we first gated single cells on a forward scatter (FSC-H)/side scatter (SSC-A) density plot. Afterward, we gated cells on a time/side scatter density plot to visualize how well the flow of cells was during acquisition. We recognized the lymphocyte population on a side scatter (SSC-A)/forward scatter (FSC-A) plot. Then, we gated lymphocytes using the CD3 expression, acquiring 10,000 events on this gate for posterior analyses. After that, we obtained CD4+ and CD8+ T cells through a rectangular gating strategy using CD4 and CD8 expression. Finally, we analyzed IFN-gamma, IL-2, and PD-1 expression on the CD4+ and CD8+ T cell populations (**Supplementary Figures 2A, B**, respectively). For monocytes, we first gated single cells on a forward scatter (FSC-H)/side scatter density plot. Then, we gated cells on a time/side scatter density plot. Afterward, we recognized monocytes using HLA-DR expression, acquiring 10,000 events on this gate for posterior analyses. Then, we obtained total monocytes on a CD14/CD16 density plot and subsequently identified gates for classical monocytes (CD14++CD16-), intermediate monocytes (CD14++CD16+), and non-classical monocytes (CD14+CD16+). Finally, we analyzed IFN-alpha expression on each monocyte subset (**Supplementary Figure 3**). We obtained the Median Fluorescence Intensity (MFI) for IL-2, IFN-gamma, PD-1, and IFN-alpha, considering both positive and negative cell populations for each marker, as shown in **Supplementary Figure 4**. We obtained the percentage of positive cells for each marker using proper fluorescence minus one (FMO) controls. We performed compensation controls for each fluorochrome by UltraComp eBeads™ (Invitrogen™, Carlsbad, CA, USA). We analyzed data using the FlowJo 10.0.7 software (TreeStar, Inc, Ashland, OR, USA).

### Total IgG and Neutralizing Antibodies Anti-SARS-CoV-2

One month after clinical recovery of the twelve patients who developed mild or severe COVID-19, we collected venous blood samples for posterior serum isolation and measurement of anti-SARS-CoV-2 IgG total antibodies and neutralizing antibody percentage in triplicate by the Enzyme Linked-ImmunoSorbent Assay (ELISA). For total antibodies, we measured IgG antibody serum levels against the SARS-CoV-2 nucleocapsid (N) protein using a kit from Abcam (Abcam, ab274339, Cambridge, UK) and a microplate reader at 450 nm. For neutralizing antibody percentage, we used the anti-SARS-CoV-2 Neutralizing Antibody ELISA Kit and a microplate reader at 450 nm (Thermo Fisher Scientific, BMS2326, Vienna, Austria). We calculated the neutralization percentage for unknown samples as follows: neutralization (%) = 1 – (absorbance of unknown sample/absorbance of negative control) × 100.

### Statistics

We evaluated the normality of data by the Shapiro-Wilk test. For *in vitro* assays, we compared the basal expression of IL-2, PD-1, IFN-gamma, and IFN-alpha between mild COVID-19 cases and severe COVID-19 cases by the unpaired Student’s T test. We compared the amount of IFN-gamma+IL-2+ double-positive cells in helper and cytotoxic T cells expressing or not PD-1 from mild COVID-19 cases and severe COVID-19 cases by two-way ANOVA. We compared the anti-SARS-CoV-2 IgG total antibodies and neutralizing antibody percentage between mild COVID-19 cases and severe COVID-19 cases by the unpaired Student’s T test. We considered differences significant when *P* < 0.05. We performed all statistical analyses using the GraphPad Prism 7 software.

## Results

### Demographic, Clinical, and Laboratory Parameters in the Study Population

**Figure 1:**
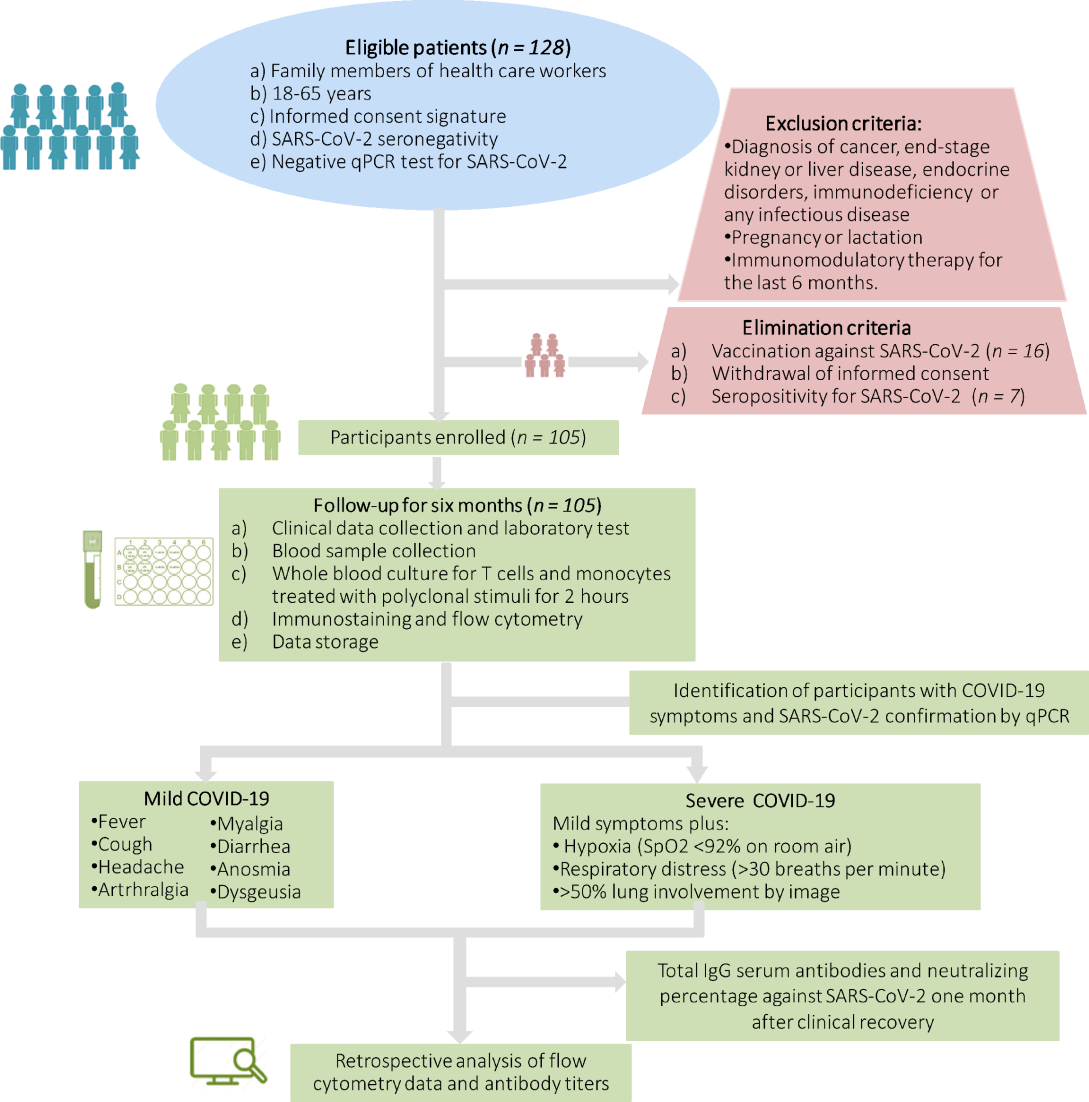
Schematic flow chart showing the selection process of eligible participants. SpO2, oxygen saturation level; SARS-CoV-2, severe acute respiratory syndrome coronavirus type 2; qPCR, quantitative polymerase chain reaction; COVID-19, coronavirus disease 2019.

We show the selection process of participants enrolled in the study in **Figure 1**. After meeting inclusion and exclusion criteria, we eliminated 23 participants from the study because they exhibited specific IgG serum antibody titers against the SARS-CoV-2 N protein (*n* = 7) or got the Pfizer-BioNTech COVID-19 vaccine (*n* = 16). One hundred and five volunteers completed the six-month follow-up without getting vaccinated (**Figure 1**). At the beginning of the follow-up, the whole study population consisted of 41 women and 64 men that were 41.6 ± 11.2 years on average and showed a low prevalence of comorbidities such as obesity, type 2 diabetes, hypertension, coronary heart disease and average values of routine biochemical tests (**Table 1**). After grading COVID-19 severity in participants who got SARS-CoV-2 infection during the six-month follow-up, we registered two women and four men only experiencing a self-limiting disease with mild symptoms such as fever, anosmia, headache, myalgia, and arthralgia, resolved without specific drug treatment after 9-12 days (**Table 2**). Conversely, one woman and five men developed severe COVID-19 characterized by the symptoms mentioned above, plus respiratory distress (36.6 ± 3.1 breaths per minute), hypoxia (74.0 ± 7.6%), and pneumonia (**Table 2**). Two participants developing severe COVID-19 required ICU admission for invasive ventilatory support (**Table 2**). Of note, the baseline clinical characteristics did not differ between participants who developed mild or severe COVID-19 during the follow-up (**Table 1**). Except for neutrophil and lymphocyte percentages, ALP, and serum albumin, there were no differences between participants who developed mild or severe COVID-19 for the values of hematic biometry, blood glucose, lipid profile, renal parameters, and liver function tests seven days after symptom onset (**Table 2**). As we have outlined here, neither baseline characteristics nor the clinical presentation of COVID-19 allowed predicting the disease severity in the participants who got SARS-CoV-2 infection during the follow-up.

**Table 1:** Baseline characteristics of the study participants.

Parameter	Baseline characteristics in the entire study population	Baseline characteristics only in participants who developed COVID-19		Reference ranges	*P* value^a vs b^
		Mild^a^	Severe^b^		
Gender (W/M)	41/64	2/4	1/5	-	0.505
Age (years)	41.6 ± 11.2	47.3 ± 10.3	45 ± 9.6	-	0.343
BMI (kg/m^2^)	27.3 ± 4.8	26 ± 1.6	25.5 ± 1.1	<25.0	0.458
Obesity (W/M)	14/10	0/0	0/1	-	1.000
D prevalence (%)	6	0	0	-	1.000
Hypertension prevalence (%)	8.6	0	0	-	1.000
Coronary heart disease (%)	1.7	0	0	-	1.000
Heart rate (beats per minute)	75.4 ± 10.8	72.5 ± 5.9	70.5 ± 5.2	60-100	0.550
Breathing rate (bpm)	15.7 ± 3.3	16.5 ± 2.5	15.6 ± 2.8	15-20	0.609
Body temperature (°C)	36.2 ± 0.2	36.1 ± 0.2	36.1 ± 0.1	≤37.5	>0.999
Systolic blood pressure (mmHg)	128.6 ± 18.3	124.5 ± 11.5	125.8 ± 7.2	<130	0.816
Diastolic blood pressure (mmHg)	82.1 ± 11.3	78.5 ± 9.3	80.1 ± 6.3	<85	0.725
Peripheral oxygen saturation (%)	95.8 ± 2.2	95.6 ± 1.0	96.1 ± 2.7	>91	0.682
Leukocytes (×10^3^/μl)	6.3 ± 1.2	6.1 ± 0.8	6.9 ± 1.8	4.00-11.00	0.213
Neutrophil percentage (%)	58.3 ± 8.6	55.2 ± 10.4	57.2 ± 7.2	37.00-80.00	0.367
Lymphocyte percentage (%)	31.5 ± 7.2	33.8 ± 9.6	27.4 ± 4.1	10.00-50.00	0.106
Monocyte percentage (%)	5.8 ± 0.9	6.3 ± 0.3	7.4 ± 1.6	0.00-8.00	0.084
Band cells (%)	0.04 ± 0.03	0.08 ± 0.08	0.00 ± 0.00	0.00-7.00	0.216
Hemoglobin (g/dl)	16.7 ± 1.0	16.2 ± 1.4	16.9 ± 0.7	13.1-18.00	0.173
Platelets (×10^3^/μl)	219.3 ± 24.6	180 ± 22.9	208.6 ± 26.5	150 -400	0.053
Glucose (mg/dl)	96.3 ± 7.4	83.4 ± 6.1	92 ± 9.7	70.0-126.0	0.447
Urea (mg/dl)	30.2 ± 8.6	28.6 ± 4.9	39.2 ± 10.5	20.00-40.00	0.059
Creatinine (mg/dl)	1.1 ± 0.2	0.9 ± 0.1	0.8 ± 0.1	0.60-1.30	0.180
Total Cholesterol (mg/dl)	207.9 ± 48.0	178.8 ± 18.3	171.4 ± 37.5	50.0-200.0	0.350
Triglycerides (mg/dl)	180.5 ± 82.6	196.8 ± 104	134.8 ± 59.1	30.0-200.0	0.140
HDL (mg/dl)	44.7 ± 8.5	40.6 ± 10.8	41 ± 9.4	≤45.00	0.475
LDL (mg/dl)	110.6 ± 34.2	114. 6 ± 33.3	117.8 ± 28.4	<116.00	0.437
Total bilirubin (mg/dl)	0.9 ± 0.1	0.7 ± 0.2	0.7 ± 0.2	0.00-1.00	0.423
Direct bilirubin (mg/dl)	0.2 ± 0.1	0.2 ± 0.1	0.1 ± 0.06	0.00-0.30	0.211
Indirect bilirubin (mg/dl)	0.5 ± 0.2	0.5 ± 0.2	0.6 ± 0.2	0.20-1.00	0.421
ALT (IU/l)	30.4 ± 8.9	27.6 ± 9.6	24 ± 9.9	30-65	0.288
AST (IU/l)	28.2 ± 5.6	23.6 ± 6.8	19.4 ± 3.6	15-37	0.129
ALP (IU/l)	51.5 ± 14.1	17.6 ± 16.8	59.2 ± 15.7	50-136	0.116
GGT (IU/l)	31.2 ± 19.7	36.4 ± 31.0	35.7 ± 17.4	5-40	0.485
Total Protein (mg/dl)	7.1 ± 0.1	7.2 ± 0.1	7.1 ± 0.2	6.50-8.20	0.275
Albumin (mg/dl)	4.8 ± 0.4	4.29 ± 0.2	4.4 ± 0.3	3.50-5.00	0.267
LDH (IU/l)	194.2 ± 17.0	179.2 ± 4.7	162.6 ± 25.0	100-190	0.118
Amylase (IU/l)	51.3 ± 15.6	45.5 ± 13.2	47.4 ± 17.1	25-115	0.430
Lipase (IU/l)	37.4 ± 14.8	28.5 ± 12.5	24.8 ± 10.8	12-70	0.324

Reference values are shown according to the Clinical Laboratory of the General Hospital of Mexico. We expressed data as mean ± standard deviation. We retrospectively compared baseline characteristics between mild and severe groups using the chi-square test or the unpaired Student’s T-test and considered differences significant when *P* < 0.05. COVID-19, coronavirus disease 2019; W, women; M, men; BMI, body mass index; D, type 2 diabetes; bpm, breaths per minute; HDL, high-density lipoproteins; LDL, low-density lipoproteins; ALT, alanine aminotransferase; AST, aspartate aminotransferase; ALP, alkaline phosphatase; GGT, gamma glutamyl transferase; LDH, lactate dehydrogenase.We show demographic, clinical, and biochemical baseline parameters in all participants and those who developed COVID-19 during the six-month follow-up.

**Table 2:** Clinical and biochemical characteristics of participants who developed mild or severe COVID-19 during the six-month follow-up.

Parameter	Onset of symptoms			*P* value ^a^ vs ^b^
	Mild COVID-19^a^	Severe COVID-19^b^	Reference ranges	
Fever (Yes/No)	4/2	5/1	<37.5°C	1.000
Anosmia (Yes/No)	4/2	3/3	-	1.000
Headache (Yes/No)	6/0	6/0	-	1.000
Myalgia (Yes/No)	4/2	5/1	-	1.000
Arthralgia (Yes/No)	2/4	1/5	-	1.000
Diarrhea (Yes/No)	3/3	2/4	-	1.000
ICU admission (Yes/No)	0/6	2/4	-	0.227
	Clinical characteristics seven days after symptom onset			
Heart rate (beats per minute)	69.1 ± 6.3	81.3 ± 8.9	60-100	0.021
Breathing rate (bpm)	19.8 ± 3.0	36.6 ± 3.1	15-20	<0.0001
Peripheral oxygen saturation (%)	95.1 ± 1.7	74.0 ± 7.6	>91	<0.0001
Leukocytes (×10^3^/μl)	5.6 ± 1.4	11.0 ± 5.6	4.00-11.00	0.066
Neutrophil percentage (%)	48.6 ± 14.2	80.1 ± 15.6	37.00-80.00	0.044*
Lymphocyte percentage (%)	40.9 ± 13.0	13.8 ± 14.8	10.00-50.00	0.043*
Monocyte percentage (%)	7.3 ± 0.1	5.8 ± 1.7	0.00-8.00	0.200
Band cells (%)	0.0 ± 0.0	0.0 ± 0.0	0.00-7.00	>0.999
Hemoglobin (g/dl)	16.7 ± 0.7	14.5 ± 1.7	13.1-18.00	0.088
Platelets (×10^3^/μl)	273.5 ± 16.2	283.4 ± 112.5	150-400	0.444
Glucose (mg/dl)	89.0 ± 12.7	208.9 ± 92.1	70.00-100.00	0.088
Urea (mg/dl)	34.8 ± 12.5	58.9 ± 68.6	20.00-40.00	0.355
Creatinine (mg/dl)	0.9 ± 0.0	1.6 ± 2.3	0.80-1.30	0.354
Total Cholesterol (mg/dl)	194.0 ± 42.4	116.7 ± 21.1	50.00-200.00	0.071
Triglycerides (mg/dl)	344.5 ± 86.9	162.8 ± 81.6	30.00-150.00	0.142
HDL (mg/dl)	32.5 ± 7.7	27.0 ± 8.2	≤45.00	0.428
LDL (mg/dl)	118.0 ± 26.8	79.3 ± 27.7	≤115.00	0.142
Total bilirubin (mg/dl)	0.8 ± 0.3	0.5 ± 0.1	0.00-1.00	0.500
Direct bilirubin (mg/dl)	0.1 ± 0.0	0.1 ± 0.0	0.00-0.30	>0.999
Indirect bilirubin (mg/dl)	0.6 ± 0.2	0.4 ± 0.1	0.20-1.00	0.500
ALT (IU/l)	37.5 ± 27.5	49.2 ± 37.8	30-65	0.711
AST (IU/l)	23.5 ± 6.3	48.5 ± 32.5	15-37	0.133
ALP (IU/l)	60.0 ± 2.8	99.3 ± 17.0	50-136	0.044*
GGT (IU/l)	46.0 ± 24.4	56.7 ± 27.3	5-40	0.755
Total Protein (mg/dl)	7.4 ± 0.0	6.7 ± 0.6	6.50-8.20	0.166
Albumin (mg/dl)	4.6 ± 0.0	3.6 ± 0.4	3.50-5.00	0.044*
LDH (IU/l)	162.5 ± 38.8	354.6 ± 146.5	100-190	0.088
Amylase (IU/l)	45.0 ± 9.8	65.0 ± 66.8	25-115	0.700
Lipase (IU/l)	41.0 ± 14.1	45.3 ± 45.6	12-70	0.810

We recorded data from all COVID-19 participants at the onset of symptoms and seven days after. Reference values are shown according to the Clinical Laboratory of the General Hospital of Mexico. We expressed data as mean ± standard deviation. We compared clinical and biochemical data between mild and severe groups using the chi-square test or the unpaired Student’s T-test and considered differences significant when P < 0.05. The asterisks represent significant differences. COVID-19, coronavirus disease 2019; ICU, intensive care unit; bpm, breaths per minute; HDL, high-density lipoproteins; LDL, low-density lipoproteins; ALT, alanine aminotransferase; AST, aspartate aminotransferase; ALP, alkaline phosphatase; GGT, gamma glutamyl transferase; LDH, lactate dehydrogenase.

### IL-2, IFN-Gamma, and PD-1 Production in Helper T Cells

**Figure 2:**
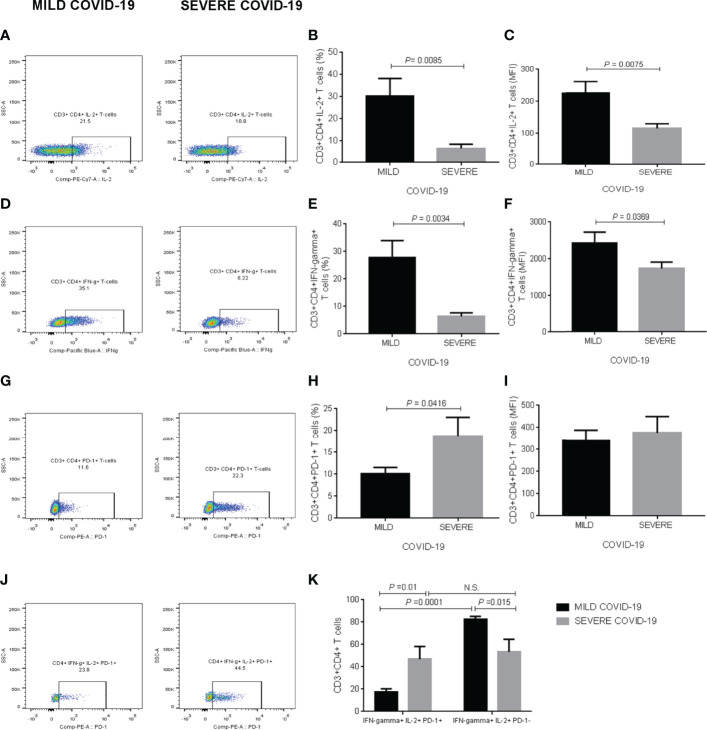
IL-2, IFN-gamma, and PD-1 expression in CD3+CD4+ T cells in response to polyclonal stimuli *in vitro*. **(A)** Representative dot-plots illustrating the comparison of CD3+CD4+ T cells expressing IL-2 in blood samples treated with polyclonal stimuli from participants who developed mild or severe COVID-19 during the follow-up. **(B)** Percentage of CD3+CD4+ T cells expressing IL-2. **(C)** Mean fluorescence intensity of IL-2 in CD3+CD4+ T cells. **(D)** Representative dot-plots exemplifying the contrast of CD3+CD4+ T cells expressing IFN-gamma in whole blood samples exposed to polyclonal molecules from participants who experienced mild or severe COVID-19 throughout the follow-up. **(E)** Percentage of CD3+CD4+ T cells expressing IFN-gamma. **(F)** Mean fluorescence intensity of IFN-gamma in CD3+CD4+ T cells. **(G)** Representative dot-plots illustrating the comparison of CD3+CD4+ T cells expressing PD-1 in blood samples treated with polyclonal stimuli from participants who developed mild or severe COVID-19 during the follow-up. **(H)** Percentage of CD3+CD4+ T cells expressing PD-1. **(I)** Mean fluorescence intensity of PD-1 in CD3+CD4+ T cells. **(J)** Representative dot-plots exemplifying the contrast of CD3+CD4+ T cells simultaneously expressing IL-2, IFN-gamma, and PD-1 in whole blood samples exposed to polyclonal molecules from participants who experienced mild or severe COVID-19 throughout the follow-up. **(K)** Percentage of CD3+CD4+ T cells producing both IL-2 and IFN-gamma depending on PD-1 expression. Prior to infection, we obtained whole blood samples from all participants enrolled in the study and cultured them with polyclonal stimuli, including anti-CD3, anti-CD28, poly I:C, and SARS-CoV-2 recombinant spike S1 protein for two hours. We then acquired CD3+CD4+ T cells on a BD FACS Canto II Flow Cytometer, acquiring 10,000 events per test in triplicate and storing data until a participant got SARS-CoV-2 infection. Upon infection and depending on the clinical course of the disease, we analyzed flow cytometry data as part of the mild or severe COVID-19 groups. Black bars represent the mild COVID-19 group. Gray bars represent the severe COVID-19 group. We expressed data as mean ± standard deviation. We compared data using the unpaired Student’s T-test or two-way ANOVA and considered differences significant when *P* < 0.05. IL-2, interleukin-2; IFN-gamma, interferon-gamma; PD-1, programmed cell death protein 1; MFI, mean fluorescence intensity; COVID-19, coronavirus disease 2019; SARS-CoV-2, severe acute respiratory syndrome coronavirus type 2.

Supplementary **Figure 2A** illustrates the gating strategy for analyzing IL-2, IFN-gamma, and PD-1 expression in CD3+CD4+ T cells stimulated with anti-CD3, anti-CD28, poly I:C, and SARS-CoV-2 recombinant spike S1 protein in whole blood *in vitro* cultures at the beginning of the follow-up, when all healthy participants were initially enrolled in the study (**Supplementary Figure 2A**). Representative dot-plots illustrate the comparison of CD3+CD4+ T cells expressing IL-2 in blood samples treated with polyclonal stimuli from participants who developed mild or severe COVID-19 during the follow-up (**Figure 2A**). In response to polyclonal *in vitro* stimulation, the percentage of CD3+CD4+IL-2+ T cells showed a significant 4-fold decrease in the group that during the follow-up developed severe COVID-19 compared to participants experiencing mild symptoms (*P* = 0.0085) (**Figure 2B**). IL-2 expression behaved in the same way as observed in cell percentage, displaying a significant 2-fold diminution in helper T cells from participants that after SARS-CoV-2 infection developed severe COVID-19 compared to the mild disease group (*P* = 0.0075) (**Figure 2C**). Representative dot-plots exemplify the contrast of CD3+CD4+ T cells expressing IFN-gamma in whole blood samples exposed to polyclonal molecules from participants who experienced mild or severe COVID-19 throughout the follow-up (**Figure 2D**). The percentage of CD3+CD4+IFN-gamma+ T cells displayed a significant 4-fold reduction in the severe COVID-19 group compared to participants developing a mild disease (*P* = 0.0034) (**Figure 2E**). Likewise, IFN-gamma production significantly decreased in helper T cells from participants experiencing severe COVID-19 compared to those found in individuals with mild symptoms (*P* = 0.0369) (**Figure 2F**). Representative dot-plots illustrate the comparison of CD3+CD4+ T cells expressing PD-1 in blood samples treated with polyclonal stimuli from participants who developed mild or severe COVID-19 during the follow-up (**Figure 2G**). The percentage of CD3+CD4+ T cells expressing PD-1 exhibited a significant 2-fold increase in the severe COVID-19 group compared to the mild disease group (*P* = 0.0416) (**Figure 2H**). There were no significant changes between severe and mild COVID-19 participants for PD-1 expression in the population of helper T cells (**Figure 2I**). Representative dot-plots exemplify the contrast of CD3+CD4+ T cells simultaneously expressing IL-2, IFN-gamma, and PD-1 in whole blood samples exposed to polyclonal molecules from participants who experienced mild or severe COVID-19 throughout the follow-up (**Figure 2J**). Interestingly, PD-1 expression was intimately related to IL-2 and IFN-gamma production in the population of helper T cells. In this sense, participants who developed mild symptoms showed that PD-1+ T cells expressing both IL-2 and IFN-gamma decreased 4-fold compared to PD-1 negative helper T lymphocytes (*P* = 0.0001), indicating that PD-1 expression inversely associated with IL-2 and IFN-gamma production (**Figure 2K**). Nevertheless, we did not observe this expected behavior in participants developing severe COVID-19 after SARS-CoV-2 infection, whose helper T cells exhibited similar IL-2 and IFN-gamma expression patterns independently of expressing or not PD-1 (**Figure 2K**). Additionally to cell percentages, we show the corresponding absolute numbers of CD3+CD4+ T cells expressing IL-2, IFN-gamma, and PD-1 in **Supplementary Figure 5**. We found no detectable IL-2, IFN-gamma, and PD-1 expression in CD3+CD4+ T cells cultured in the absence of anti-CD3, anti-CD28, poly I:C, and SARS-CoV-2 recombinant spike S1 protein (data not shown).

### IL-2, IFN-Gamma, and PD-1 Production in Cytotoxic T Cells

**Figure 3:**
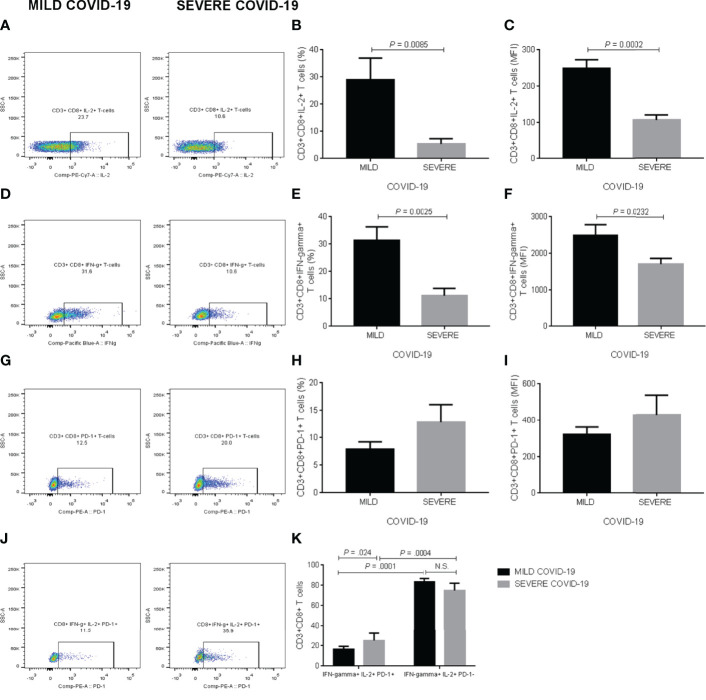
IL-2, IFN-gamma, and PD-1 expression in CD3+CD8+ T cells in response to polyclonal stimuli *in vitro*. **(A)** Representative dot-plots illustrating the comparison of CD3+CD8+ T cells expressing IL-2 in blood samples treated with polyclonal stimuli from participants who developed mild or severe COVID-19 during the follow-up. **(B)** Percentage of CD3+CD8+ T cells expressing IL-2. **(C)** Mean fluorescence intensity of IL-2 in CD3+CD8+ T cells. **(D)** Representative dot-plots exemplifying the contrast of CD3+CD8+ T cells expressing IFN-gamma in whole blood samples exposed to polyclonal molecules from participants who experienced mild or severe COVID-19 throughout the follow-up. **(E)** Percentage of CD3+CD8+ T cells expressing IFN-gamma. **(F)** Mean fluorescence intensity of IFN-gamma in CD3+CD8+ T cells. **(G)** Representative dot-plots illustrating the comparison of CD3+CD8+ T cells expressing PD-1 in blood samples treated with polyclonal stimuli from participants who developed mild or severe COVID-19 during the follow-up. **(H)** Percentage of CD3+CD8+ T cells expressing PD-1. **(I)** Mean fluorescence intensity of PD-1 in CD3+CD8+ T cells. **(J)** Representative dot-plots exemplifying the contrast of CD3+CD8+ T cells simultaneously expressing IL-2, IFN-gamma, and PD-1 in whole blood samples exposed to polyclonal molecules from participants who experienced mild or severe COVID-19 throughout the follow-up. **(K)** Percentage of CD3+CD8+ T cells producing both IL-2 and IFN-gamma depending on PD-1 expression. Prior to infection, we obtained whole blood samples from all participants enrolled in the study and cultured them with polyclonal stimuli, including anti-CD3, anti-CD28, poly I:C, and SARS-CoV-2 recombinant spike S1 protein for two hours. We then acquired CD3+CD8+ T cells on a BD FACS Canto II Flow Cytometer, acquiring 10,000 events per test in triplicate and storing data until a participant got SARS-CoV-2 infection. Upon infection and depending on the clinical course of the disease, we analyzed flow cytometry data as part of the mild or severe COVID-19 groups. Black bars represent the mild COVID-19 group. Gray bars represent the severe COVID-19 group. We expressed data as mean ± standard deviation. We compared data using the unpaired Student’s T-test or two-way ANOVA and considered differences significant when *P* < 0.05. IL-2, interleukin-2; IFN-gamma, interferon-gamma; PD-1, programmed cell death protein 1; MFI, mean fluorescence intensity; COVID-19, coronavirus disease 2019; SARS-CoV-2, severe acute respiratory syndrome coronavirus type 2.

Supplementary figure 2B shows the gating strategy for examining IL-2, IFN-gamma, and PD-1 expression in CD3+CD8+ T cells exposed to polyclonal stimuli *in vitro* (**Supplementary Figure 2B**). Representative dot-plots illustrate the comparison of CD3+CD8+ T cells expressing IL-2 in blood samples treated with polyclonal stimuli from participants who developed mild or severe COVID-19 during the follow-up (**Figure 3A**). In response to anti-CD3, anti-CD28, poly I:C, and SARS-CoV-2 recombinant spike S1 protein, whole blood cultures revealed that the percentage of CD3+CD8+IL-2+ T cells exhibited a significant 5-fold decrease in the group that posteriorly developed severe COVID-19 compared to participants with mild symptoms (*P* = 0.0085) (**Figure 3B**). As expected, IL-2 production significantly reduced in cytotoxic T cells from participants developing severe COVID-19 compared to those found in subjects experiencing mild disease (*P* = 0.0002) (**Figure 3C**). Representative dot-plots exemplify the contrast of CD3+CD8+ T cells expressing IFN-gamma in whole blood samples exposed to polyclonal molecules from participants who experienced mild or severe COVID-19 throughout the follow-up (**Figure 3D**). The percentage of CD3+CD8+IFN-gamma+ T cells showed a significant 3-fold diminution in the severe COVID-19 group compared to participants experiencing mild symptoms (*P* = 0.0025) (**Figure 3E**). In parallel, IFN-gamma expression significantly decreased in the cytotoxic T cell population of participants who developed severe COVID-19 compared to that found in subjects with mild symptoms (*P* = 0.0232) (**Figure 3F**). Representative dot-plots illustrate the comparison of CD3+CD8+ T cells expressing PD-1 in blood samples treated with polyclonal stimuli from participants who developed mild or severe COVID-19 during the follow-up (**Figure 3G**). Contrary to what we expected, neither the percentage of CD3+CD8+PD-1+ T cells nor PD-1 production itself exhibited significant differences between the mild and severe COVID-19 groups (**Figures 3H, I**, respectively). Representative dot-plots exemplify the contrast of CD3+CD8+ T cells simultaneously expressing IL-2, IFN-gamma, and PD-1 in whole blood samples exposed to polyclonal molecules from participants who experienced mild or severe COVID-19 throughout the follow-up (**Figure 3J**). However, PD-1 expression conditioned IL-2 and IFN-gamma production in cytotoxic T cells, which expressed higher IL-2 and IFN-gamma levels in PD-1 negative cells than CD3+CD8+PD-1+ T cells independently of having been analyzed in participants that posteriorly developed either mild or severe COVID-19 (**Figure 3K**). In addition to cell percentages, we provide the corresponding absolute numbers of CD3+CD8+ T cells expressing IL-2, IFN-gamma, and PD-1 in **Supplementary Figure 5**. We observed no detectable IL-2, IFN-gamma, and PD-1 expression levels in CD3+CD8+ T cells cultured without anti-CD3, anti-CD28, poly I:C, and recombinant spike S1 protein (data not shown).

### IFN-Alpha Production in Monocyte Subpopulations

**Figure 4:**
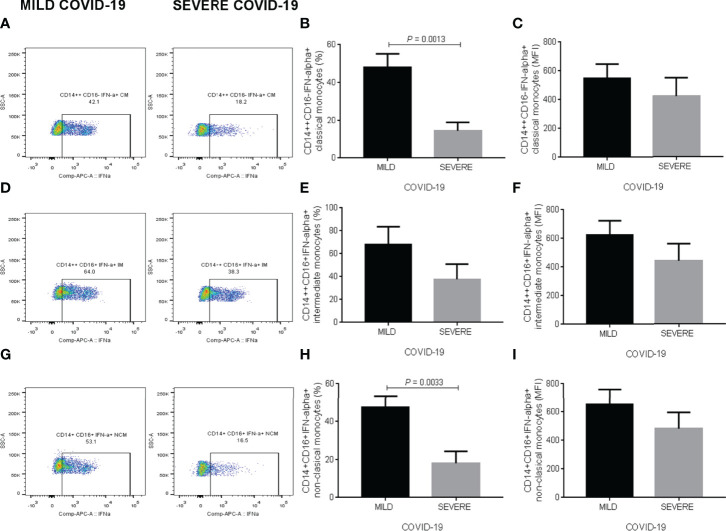
IFN-alpha expression in classical, intermediate, and non-classical monocyte subpopulations in response to polyclonal stimuli *in vitro*. **(A)** Representative dot-plots illustrating the comparison of CD14++CD16- classical monocytes expressing IFN-alpha in blood samples treated with polyclonal stimuli from participants who developed mild or severe COVID-19 during the follow-up. **(B)** Percentage of CD14++CD16- classical monocytes expressing IFN-alpha. **(C)** Mean fluorescence intensity of IFN-alpha in CD14++CD16- classical monocytes. **(D)** Representative dot-plots exemplifying the contrast of CD14++CD16+ intermediate monocytes expressing IFN-alpha in whole blood samples exposed to polyclonal molecules from participants who experienced mild or severe COVID-19 throughout the follow-up. **(E)** Percentage of CD14++CD16+ intermediate monocytes expressing IFN-alpha. **(F)** Mean fluorescence intensity of IFN-alpha in CD14++CD16+ intermediate monocytes. **(G)** Representative dot-plots illustrating the comparison of CD14+CD16+ non-classical monocytes expressing IFN-alpha in blood samples treated with polyclonal stimuli from participants who developed mild or severe COVID-19 during the follow-up. **(H)** Percentage of CD14+CD16+ non-classical monocytes expressing IFN-alpha. **(I)** Mean fluorescence intensity of IFN-alpha in CD14+CD16+ non-classical monocytes. Prior to infection, we obtained whole blood samples from all participants enrolled in the study and cultured them with polyclonal stimuli, including poly I:C, SARS-CoV-2 recombinant spike S1 protein, and LPS, for two hours. We then acquired monocyte cells on a BD FACS Canto II Flow Cytometer, acquiring 10,000 events per test in triplicate and storing data until a participant got SARS-CoV-2 infection. Upon infection and depending on the clinical course of the disease, we analyzed flow cytometry data as part of the mild or severe COVID-19 groups. Black bars represent the mild COVID-19 group. Gray bars represent the severe COVID-19 group. We expressed data as mean ± standard deviation. We compared data using the unpaired Student’s T-test and considered differences significant when *P* < 0.05. We defined classical monocytes as CD14++CD16-, intermediate monocytes as CD14++CD16+, and non-classical monocytes as CD14+CD16+. IFN-alpha, interferon-alpha; MFI, mean fluorescence intensity; COVID-19, coronavirus disease 2019, LPS, lipopolysaccharide; SARS-CoV-2, severe acute respiratory syndrome coronavirus type 2.

**Supplementary Figure 3** illustrates the gating strategy for evaluating IFN-alpha expression in classical, intermediate, and non-classical monocytes exposed to SARS-CoV-2 recombinant spike S1 protein, poly I:C and LPS in whole blood *in vitro* cultures (**Supplementary Figure 3**). Representative dot-plots illustrate the comparison of CD14++CD16- classical monocytes expressing IFN-alpha in blood samples treated with polyclonal stimuli from participants who developed mild or severe COVID-19 during the follow-up (**Figure 4A**). In response to polyclonal *in vitro* stimulation, the percentage of classical monocytes expressing IFN-alpha showed a significant 3-fold decrease in the group who developed severe COVID-19 during the follow-up compared to participants with mild symptoms (*P* = 0.0013) (**Figure 4B**). There were no significant changes between mild and severe COVID-19 group for IFN-alpha expression in this monocyte subset (**Figure 4C**). Representative dot-plots exemplify the contrast of CD14++CD16+ intermediate monocytes expressing IFN-alpha in whole blood samples exposed to polyclonal molecules from participants who experienced mild or severe COVID-19 throughout the follow-up (**Figure 4D**). Neither the percentage of intermediate monocytes expressing IFN-alpha nor IFN-alpha production itself displayed significant differences between the mild and severe COVID-19 groups (**Figures 4E, F**, respectively). Representative dot-plots illustrate the comparison of CD14+CD16+ non-classical monocytes expressing IFN-alpha in blood samples treated with polyclonal stimuli from participants who developed mild or severe COVID-19 during the follow-up (**Figure 4G**). The percentage of non-classical monocytes expressing IFN-alpha exhibited a significant 2.5-fold reduction in participants developing severe COVID-19 compared to that found in individuals experiencing mild symptoms (*P* = 0.0033) (**Figure 4H**). Conversely, there were no significant changes between mild and severe COVID-19 groups for IFN-alpha expression in this monocyte subset (**Figure 4I**). Besides showing cell percentages, we present the corresponding absolute numbers of classical, intermediate, and non-classical monocytes expressing IFN-alpha in **Supplementary Figure 5**. We found no detectable IFN-alpha expression in classical, intermediate, and non-classical monocytes cultured without recombinant spike S1 protein, poly I:C, and LPS (data not shown).

### Total IgG and Neutralizing Antibodies Anti-SARS-CoV-2

**Figure 5:**
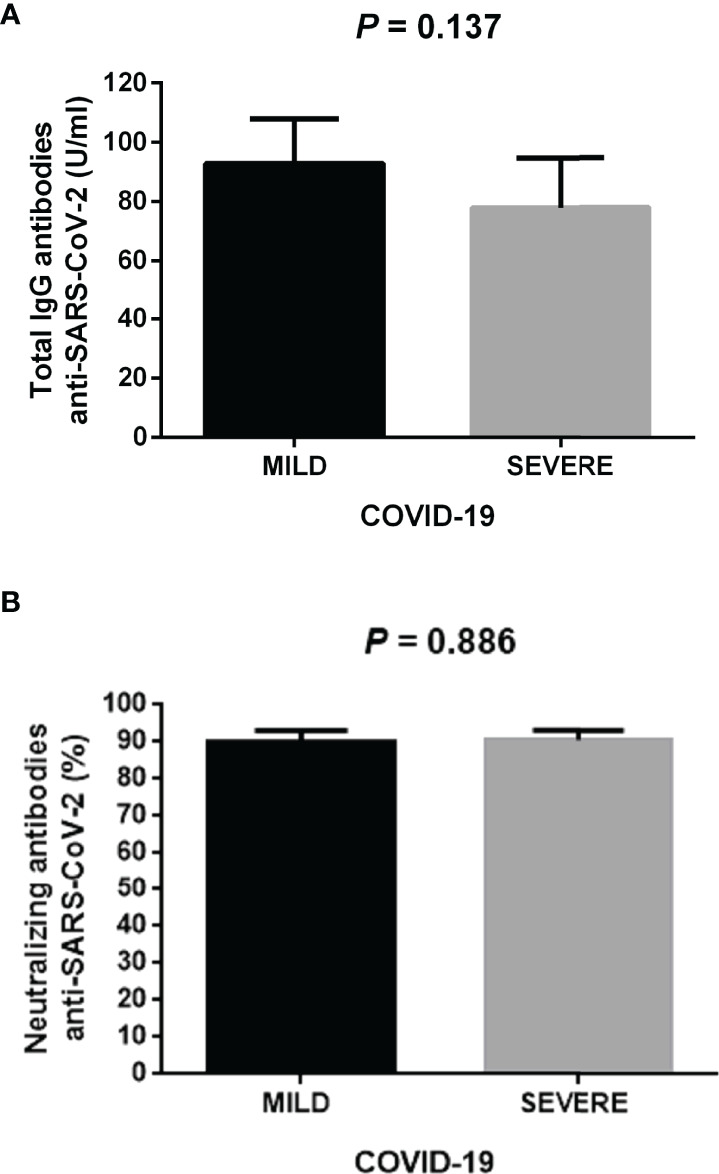
Total IgG and neutralizing antibodies anti-SARS-CoV-2 in the study participants. **(A)** Total IgG serum antibodies against the N protein of the SARS-CoV-2 in participants who developed mild or severe COVID-19 during the follow-up. **(B)** Percentage of neutralizing antibodies anti-SARS-CoV-2 in participants who developed mild or severe COVID-19 during the follow-up. We measured total IgG or neutralizing antibodies one month after clinical recovery of participants. Black bars represent the mild COVID-19 group. Gray bars represent the severe COVID-19 group. We expressed data as mean ± standard deviation. We compared data using the unpaired Student’s T-test and considered differences significant when *P* < 0.05. IgG, immunoglobulin G; SARS-CoV-2, severe acute respiratory syndrome coronavirus type 2; COVID-19, coronavirus disease 2019.

The production pattern of IL-2, IFN-gamma, and IFN-alpha suggested that helper and cytotoxic T cells and monocyte subpopulations show a basal defective cellular response against polyclonal stimuli, which is probably associated with predisposing to the development of severe COVID-19 after SARS-CoV-2 infection. To know whether a possible impairment in the cellular immune response led to defective antibody production, we decided to measure the total concentration of IgG serum antibodies and the percentage of neutralizing antibodies against the SARS-CoV-2 in participants who developed either mild or severe COVID-19. Unexpectedly, the total IgG serum titers against the N protein of the SARS-CoV-2 showed no significant changes between participants developing mild or severe COVID-19 $\left(\bar{\text{x}}=92.8\pm 15.1\text{ and }\bar{\text{x}}=\;77.9\pm 16.8,P=0.137,\text{ respectively}\right)$ (**Figure 5A**). Likewise, the neutralizing antibody percentage against the virus did not differ between participants who developed mild or severe COVID-19 $\left(\bar{\text{x}}=\;89.6\pm 7.1\text{ and }\bar{\text{x}}=\;90.2\pm 6.4,\text{ respectively},P=\;0.886\right)$ (**Figure 5B**). Thus, defective expression of IL-2, IFN-gamma, and IFN-alpha in T lymphocytes and monocytes did not affect the production of either total IgG or neutralizing antibodies against the SARS-CoV-2.

## Discussion

The mechanisms of the cell-mediated immunity contributing to worsening the severity of COVID-19 remain unclear (17, 18). For this reason, we formed a cohort of healthy individuals who were family members of health care professionals working at a dedicated COVID-19 hospital and obtained more than 120 whole blood samples. Then, we exposed T cells and monocytes to polyclonal stimuli *in vitro* to characterize a cytokine response pattern that we could link to the severity of COVID-19 only in those participants resulting infected during a six-month follow-up. We found that participants who got infected and posteriorly experienced mild COVID-19 symptoms exhibit a different immune expression pattern in response to polyclonal stimuli than that observed in subjects that developed severe COVID-19. Participants that responded to polyclonal stimuli by increasing IL-2 and IFN-gamma production and decreasing PD-1 expression in CD4+ and CD8+ T cells tended to develop mild COVID-19 symptoms. Conversely, subjects with decreased IL-2 and IFN-gamma expression and increased PD-1 production in CD4+ and CD8+ T cells in response to polyclonal stimuli tended to display the most severe form of COVID-19, including respiratory distress and mechanical ventilatory support needing. These findings reveal a basal immune response pattern to polyclonal stimuli intimately associated with COVID-19 progression, wherein CD4+ and CD8+ T cells fail to produce IL-2 and IFN-gamma but show an increased ability to express PD-1.

At the pandemic’s beginning, most studies informed that more than 80% of patients seriously ill with COVID-19 tended to exhibit a marked lymphocytopenia at hospital admission (19–21). After that, several lines of evidence confirmed that severe COVID-19 was not only related to a reduced number of circulating lymphocytes but also decreased T cell activity, especially cytokine production such as IL-2 and IFN-gamma (22, 23). IL-2 plays a decisive role in preventing lymphocytopenia by promoting CD4+ T cell proliferation *via* the Janus kinase 1/Signal transducer and activator of transcription 5 (JAK1/STAT5) (21, 24). In fact, the use of recombinant IL-2 stimulates lymphocyte count recovery and systemic inflammatory response amelioration in patients with severe COVID-19 pneumonia (25). This body of evidence makes it feasible to think that subjects with a low number of IL-2-producing CD4+ T cells in response to *in vitro* polyclonal stimuli display increased susceptibility to severe COVID-19 after SARS-CoV-2 infection. IFN-gamma’s primary function in the anti-viral response is acting directly on CD8+ T cells to boost their abundance and reduce viral load (26). IFN-gamma-producing CD8+ T cells considerably decrease in patients with severe COVID-19 compared to patients experiencing mild symptoms (27). What is also relevant is that IFN-gamma is considered an independent risk factor of mortality in COVID-19 patients (28). All this information concurs with our findings and supports the idea that a basal deficiency in IFN-gamma-producing CD8+ T cells, as revealed when we used unspecific polyclonal stimuli, may increase the risk of exacerbating viral load and developing severe COVID-19 after SARS-CoV-2 infection. However, CD8+ T cells are not only able to secrete IFN-gamma but also molecules with potent cytotoxic activity such as granzyme and perforin, which are crucial components in the recognition and lysis of infected cells. Therefore, we still must characterize the production of cytotoxic molecules in CD8+ T cells exposed to polyclonal stimuli to draw significant conclusions regarding the possible role of the cytotoxic activity of CD8+ T lymphocytes in COVID-19 progression.

PD-1 has a pivotal role in preventing exacerbation of immune responses by modulating the activity of T cells *via* apoptosis promotion, cell proliferation arrest, and cytokine secretion inhibition (29). In COVID-19, the function of PD-1 is still a matter of debate because some research teams have consistently reported that CD4+ and CD8+ T cells from COVID-19 patients express high PD-1 levels and are exhausted (13, 30). In contrast, other working groups have informed that cytotoxic T cells retain their anti-viral functions against the SARS-CoV-2 despite expressing PD-1 (31). We may attribute these controversial findings to the fact that most investigations assessing PD-1 expression in COVID-19 have studied patients treated with several drug cocktails, including cyclooxygenase (COX)-inhibitors, dexamethasone, anticoagulants, among others (32, 33). These drug schemes aim to treat and prevent COVID-19 complications but can also alter the expression of cytokines such as IL-2 and IFN-gamma and immune checkpoints as occurs with PD-1. For instance, Kailin Xing and colleagues previously demonstrated that dexamethasone increases PD-1 expression and decreases IL-2 and IFN-gamma production in human primary T cells in a dose-dependent fashion (34). Likewise, celecoxib and aspirin, two COX-inhibitors widely used in COVID-19 patients, can increase PD-1 expression in CD4+ and CD8+ chimeric antigen receptor T cells *in vitro* (35). This information illustrates why trying to clarify the contribution of immune cells and mediators such as CD4+ T cells, CD8+ T cells, IL-2, IFN-gamma, and PD-1 to COVID-19 progression is extremely hard in polytreated patients already hospitalized. From a different perspective, our strategy involving unspecific polyclonal stimuli prior to infection allows us to expand on the body of evidence supporting that PD-1 expression increases as IL-2 and IFN-gamma production decreases in severe COVID-19. In other words, our results suggest that a group of individuals may have CD4+ and CD8+ T cells with a basal predisposition to express high PD-1 levels and low IL-2 and IFN-gamma amounts in response to either polyclonal stimuli or SARS-CoV-2. This notion might partially explain why participants that showed helper and cytotoxic T cells with increased PD-1 expression and decreased IL-2 and IFN-gamma production in response to polyclonal stimuli tended to develop severe COVID-19 once infected. Furthermore, these findings support the idea that failure in mounting an adequate T cell-mediated immune response at the beginning of the SARS-CoV-2 infection is associated with increased viral load, systemic inflammatory response occurrence, and death (36, 37). The molecular mechanisms behind this intriguing hypothesis remain to be elucidated, which will positively contribute to expanding our knowledge regarding the very heterogeneous immune responses of humans to pathogens, above all if they are emerging public threats as occurred with SARS-CoV-2.

Besides the response mediated by CD4+ and CD8+ T cells, monocyte subpopulations play a crucial role in the anti-viral immune response by providing the first cell-virus interaction that will lead to antigen presentation and cytokine release (38). Several research teams have shown that monocyte subsets display dynamic changes in COVID-19, including an increase in classical and non-classical monocyte subpopulations and impaired cell ability to express cytokines with anti-viral functions (16, 39, 40). In response to polyclonal stimuli, we did not observe any alteration in the monocyte subset balance; however, we found that classical and non-classical monocytes lost their ability to produce IFN-alpha in subjects that once infected developed severe COVID-19. A study conducted in COVID-19 patients reported that IFN-alpha serum levels considerably decreased as the severity of the disease increased (16). Vanessa Chilunda and coworkers characterized the transcriptional profile of CD16+ monocyte subsets from COVID-19 patients. They informed that intermediate and non-classical monocytes exhibited down-regulation of numerous interferon response-related genes in severe cases compared to subjects that experienced the mild disease (41). In line with these reports, our findings indicate that an apparent susceptibility of classical and non-classical monocytes to express low IFN-alpha levels in response to polyclonal stimuli might be associated with a higher risk of developing severe COVID-19 after SARS-CoV-2 exposure.

Cellular immunity mediated by monocytes and T cells provides the first immediate response to pathogens *via* antigen presentation and cytokine release while stimulating B cells to initiate humoral immunity through antibody production. Often, a defective cellular immunity leads to decreased memory B cell expansion and impaired antibody production, as occurs with H1N1/09 influenza vaccine non-responders where failure in CD4+ T cell stimulation and IL-2 secretion concurs with a low percentage of IgG antibody-secreting cells (42). However, the apparent link between cellular and humoral immune responses is still not clear in COVID-19. A recent study reported that PBMCs from severe COVID-19 patients show less CD4+ and CD8+ T cell activation and IFN-gamma production than PBMCs from mild cases in response to *in vitro* stimulation with M, N, and S viral proteins (43). Nevertheless, the neutralizing ability of anti-SARS-CoV-2-specific antibodies remained the same between severe and mild COVID-19 patients after a month of having been diagnosed by PCR test (43). Likewise, Irene Cassaniti and colleagues informed that CD4+ and CD8+ T cells from mild COVID-19 patients produce higher IFN-gamma concentrations than those found in T cells from severe cases in response to viral peptides (44). However, the authors reported no correlation between the *in vitro* T cell response and anti-SARS-CoV-2 antibody titers (44). In line with this body of evidence, we observed a group of subjects with a robust cellular immunity mediated by T cells and monocytes in response to polyclonal stimuli. This immune response pattern concurred with the development of mild COVID-19 symptoms after SARS-CoV-2 exposure. Conversely, we found another group of individuals that responded to polyclonal stimuli by showing a defective cellular immune activation associated with the development of severe COVID-19 once infection took place. Of note, we detected no changes between mild and severe COVID-19 patients for serum anti-SARS-CoV-2 IgG antibody titers or their neutralizing ability after a month of the symptom onset. Altogether, these findings lead us to suppose that a basal impairment in cellular immunity activation may play a more critical role in preventing COVID-19 worsening than the humoral response mediated by antibodies. We are now working on characterizing the possible mechanisms involved in stimulating PD-1 expression and impairing IL-2 and IFN-gamma production in CD4+ and CD8+ T cells and IFN-alpha secretion in classical and non-classical monocyte subsets, including differential methylation patterns and polymorphic variants.

Finally, we found a SARS-CoV-2 infection rate of around 11% in our study population, among who 50% developed a severe form of COVID-19. The Mexican government officially reported an accumulated number of SARS-CoV-2 positive cases of 725,346 for Mexico City from December 2020 to September 2021 (https://datos.covid-19.conacyt.mx). The official number of inhabitants in Mexico City was around 9,209,944 in 2021. These numbers suggest that about 8% of the general population living in Mexico City got SARS-CoV-2 infection when we conducted the study. Moreover, a recent study indicated that nearly 39% of SARS-CoV-2 positive cases in Mexico were hospitalized due to the COVID-19 severity (45). These data reflect, to some extent, what we found in our study if we consider the limited number of SARS-CoV-2 detection tests available during that period and the remarkable underestimation of the most severe COVID-19 cases in Mexico.

In conclusion, using *in vitro* polyclonal stimuli, we found two basal immune response patterns associated with a predisposition to developing mild or severe COVID-19 once SARS-CoV-2 infection occurs. The pattern linked to severe COVID-19 is characterized by high PD-1 expression, low IL-2 and IFN-gamma production in CD4+ and CD8+ T cells, and poor IFN-alpha expression in classical and non-classical monocytes. Conversely, low PD-1 synthesis and high IL-2 and IFN-gamma expression in helper and cytotoxic T cells and an increased IFN-alpha production in classical and non-classical monocyte subsets are related to a basal predisposition to developing mild COVID-19 symptoms after SARS-CoV-2 exposure. Since the serum anti-SARS-CoV-2 IgG antibody titers or their neutralizing ability did not differ between mild and severe COVID-19 cases, these findings suggest that cellular immunity may play a more crucial function than humoral immunity in preventing COVID-19 progression.

## Data Availability Statement

The raw data supporting the conclusions of this article will be made available by the authors, without undue reservation.

## Ethics Statement

The studies involving human participants were reviewed and approved by Institutional ethical committee of the General Hospital of Mexico (registration number of the ethical code approval: DI/20/501/03/17). The patients/participants provided their written informed consent to participate in this study.

## Author Contributions

Conceptualization, GE; Methodology, RV-S, AM-R, HS-V, SR-T, LM-G, VV-S, JG-S, AG-C, JIL-P, RF-M, and OR-C; Patient Enrolment, RV-S, SR-T, LM-G, VV-S, JG-S, AA-V, JC-R, AG-C, and JL-P; DATA CURATION, RV-S, AM-R, HS-V, SR-T, LM-G, VV-S, JG-S, AA-V, and JC-R; Formal Analysis, RV-S, AA-V, JC-R, AG-C, JL-P, RF-M, OR-C, and GE; Writing—Original Draft Preparation, RV-S and GE; Writing—Review And Editing, RV-S, AM-R, HS-V, SR-T, LM-G, VV-S, JS-G, AA-V, JC-R, AG-C, JL-P, RF-M, and OR-C; Funding Acquisition, GE. All authors contributed to the article and approved the submitted version.

## Funding

This work was supported by grants no. CB-2016-286209 from the Fondo Sectorial de Investigación para la Educación SEP-CONACYT-México and SALUD-2017-02-290345 from the Fondo Sectorial de Investigación y Desarrollo en Salud y Seguridad Social SS/IMSS/ISSSTE/CONACYT-México to GE.

## Conflict of Interest

The authors declare that the research was conducted in the absence of any commercial or financial relationships that could be construed as a potential conflict of interest.

## Publisher’s Note

All claims expressed in this article are solely those of the authors and do not necessarily represent those of their affiliated organizations, or those of the publisher, the editors and the reviewers. Any product that may be evaluated in this article, or claim that may be made by its manufacturer, is not guaranteed or endorsed by the publisher.

## Supplementary Material

Supplementary Figure 1 Time-response curves. **(A)** Based on time-response curves, we selected the earliest *in vitro* culture time to detect IL-2 production in CD3+ T cells. IL-2 production peaked after 2 hours on *in vitro* culture and showed no significant differences at 6, 12, and 24 hours. **(B)** Based on time-response curves, we selected the earliest *in vitro* culture time to detect IFN-gamma production in CD3+ T cells. IFN-gamma production peaked after 2 hours on *in vitro* culture and showed no significant differences at 6, 12, and 24 hours. **(C)** Based on time-response curves, we selected the earliest *in vitro* culture time to detect IFN-alpha production in CD14+ monocytes. IFN-alpha production peaked after 2 hours on *in vitro* culture and showed no significant differences at 6, 12, and 24 hours. We expressed data as mean ± standard deviation. We compared the production of IL-2, IFN-gamma, and IFN-alpha at 0, 1, 2, 6, 12, and 24 hours using one-way ANOVA and considered differences significant if *P* < 0.05. IL-2, interleukin-2; IFN-gamma, interferon-gamma; IFN-alpha, interferon-alpha; MFI, mean fluorescence intensity.

Supplementary Figure 2 Gating strategy for T cells. **(A)** Gating strategy for selecting primary human CD3+CD4+ lymphocyte subsets and measuring IL-2, IFN-gamma, and PD-1 precisely. **(B)** Gating strategy for selecting primary human CD3+CD8+ lymphocyte subsets and measuring IL-2, IFN-gamma, and PD-1 precisely. FSC-H, forward scatter height; FSC-A, forward scatter area; SSC-A, side scatter area; IL-2, interleukin-2; IFN-gamma, interferon-gamma; PD-1, programmed cell death protein 1.

Supplementary Figure 3 Gating strategy for monocyte subsets. Gating strategy for selecting primary human HLA-DR+ monocyte subsets and measuring IFN-alpha precisely. FSC-H, forward scatter height; FSC-A, forward scatter area; SSC-A, side scatter area; HLA-DR, human leukocyte antigen-DR isotype; IFN-alpha.

Supplementary Figure 4 Representative spectra of mean fluorescence intensity for IL-2, IFN-gamma, PD-1, and IFN-alpha in T cells and monocyte subsets. **(A)** Representative histograms show the comparison of CD3+CD4+ T cells expressing IL-2 in blood samples treated with polyclonal stimuli from participants who developed mild or severe COVID-19 during the follow-up. **(B)** Representative histograms show the comparison of CD3+CD8+ T cells expressing IL-2 in blood samples treated with polyclonal stimuli from participants who developed mild or severe COVID-19 throughout the follow-up. **(C)** Representative histograms show the comparison of CD3+CD4+ T cells expressing IFN-gamma in blood samples treated with polyclonal stimuli from participants who developed mild or severe COVID-19 during the follow-up. **(D)** Representative histograms show the comparison of CD3+CD8+ T cells expressing IFN-gamma in blood samples treated with polyclonal stimuli from participants who developed mild or severe COVID-19 throughout the follow-up. **(E)** Representative histograms show the comparison of CD3+CD4+ T cells expressing PD-1 in blood samples treated with polyclonal stimuli from participants who developed mild or severe COVID-19 during the follow-up. **(F)** Representative histograms show the comparison of CD3+CD8+ T cells expressing PD-1 in blood samples treated with polyclonal stimuli from participants who developed mild or severe COVID-19 throughout the follow-up. **(G)** Representative histograms show the comparison of CD14++CD16- classical monocytes expressing IFN-alpha in blood samples treated with polyclonal stimuli from participants who developed mild or severe COVID-19 throughout the follow-up. **(H)** Representative histograms show the comparison of CD14++CD16+ intermediate monocytes expressing IFN-alpha in blood samples treated with polyclonal stimuli from participants who developed mild or severe COVID-19 during the follow-up. **(I)** Representative histograms show the comparison of CD14+CD16+ non-classical monocytes expressing IFN-alpha in blood samples treated with polyclonal stimuli from participants who developed mild or severe COVID-19 throughout the follow-up. In all cases, control indicates the spectra of MFI from unstimulated cells. IL-2, interleukin-2; IFN-gamma, interferon-gamma; PD-1, programmed cell death protein 1; IFN-alpha, interferon-alpha; MFI, mean fluorescence intensity; COVID-19, coronavirus disease 2019.

Supplementary Figure 5 Absolute cell numbers for percentages of T cells and monocyte subsets. We show absolute cell numbers for percentages of CD3+CD4+ T cells expressing IL-2, IFN-gamma, and PD-1 on top. We show absolute cell numbers for percentages of CD3+CD8+ T cells expressing IL-2, IFN-gamma, and PD-1 in the middle. We show absolute cell numbers for percentages of classical, intermediate, and non-classical monocytes expressing IFN-alpha on the bottom. We defined classical monocytes as CD14++CD16-, intermediate monocytes as CD14++CD16+, and non-classical monocytes as CD14+CD16+. We expressed data as mean ± standard deviation. We compared data using the unpaired Student’s T-test and considered differences significant when *P* < 0.05. IL-2, interleukin-2; IFN-gamma, interferon-gamma; PD-1, programmed cell death protein 1; IFN-alpha, interferon-alpha; COVID-19, coronavirus disease 2019.
