# Supplementary figures and images for: In Vitro Exposure of Primary Human T Cells and Monocytes to Polyclonal Stimuli Reveals a Basal Susceptibility to Display an Impaired Cellular Immune Response and Develop Severe COVID-19
Source: Front Immunol. 2022 Jul 1;13:897995. doi: 10.3389/fimmu.2022.897995 (PMC9289744; doi:10.3389/fimmu.2022.897995)

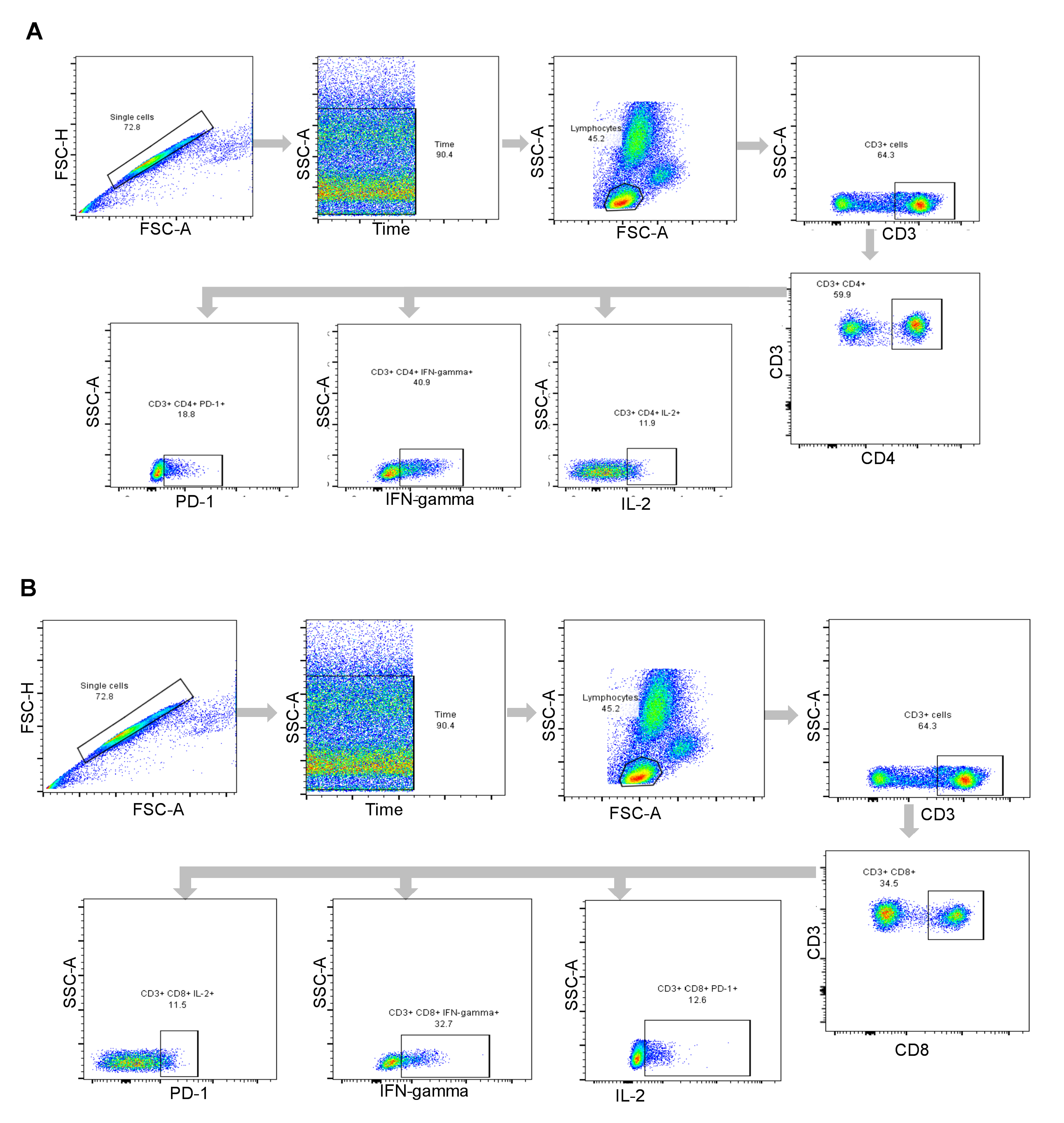

Supplement: Supplementary Figure 2 — Gating strategy for T cells. (A) Gating strategy for selecting primary human CD3+CD4+ lymphocyte subsets and measuring IL-2, IFN-gamma, and PD-1 precisely. (B) Gating strategy for selecting primary human CD3+CD8+ lymphocyte subsets and measuring IL-2, IFN-gamma, and PD-1 precisely. FSC-H, forward scatter height; FSC-A, forward scatter area; SSC-A, side scatter area; IL-2, interleukin-2; IFN-gamma, interferon-gamma; PD-1, programmed cell death protein 1. [file Image_2.tif]

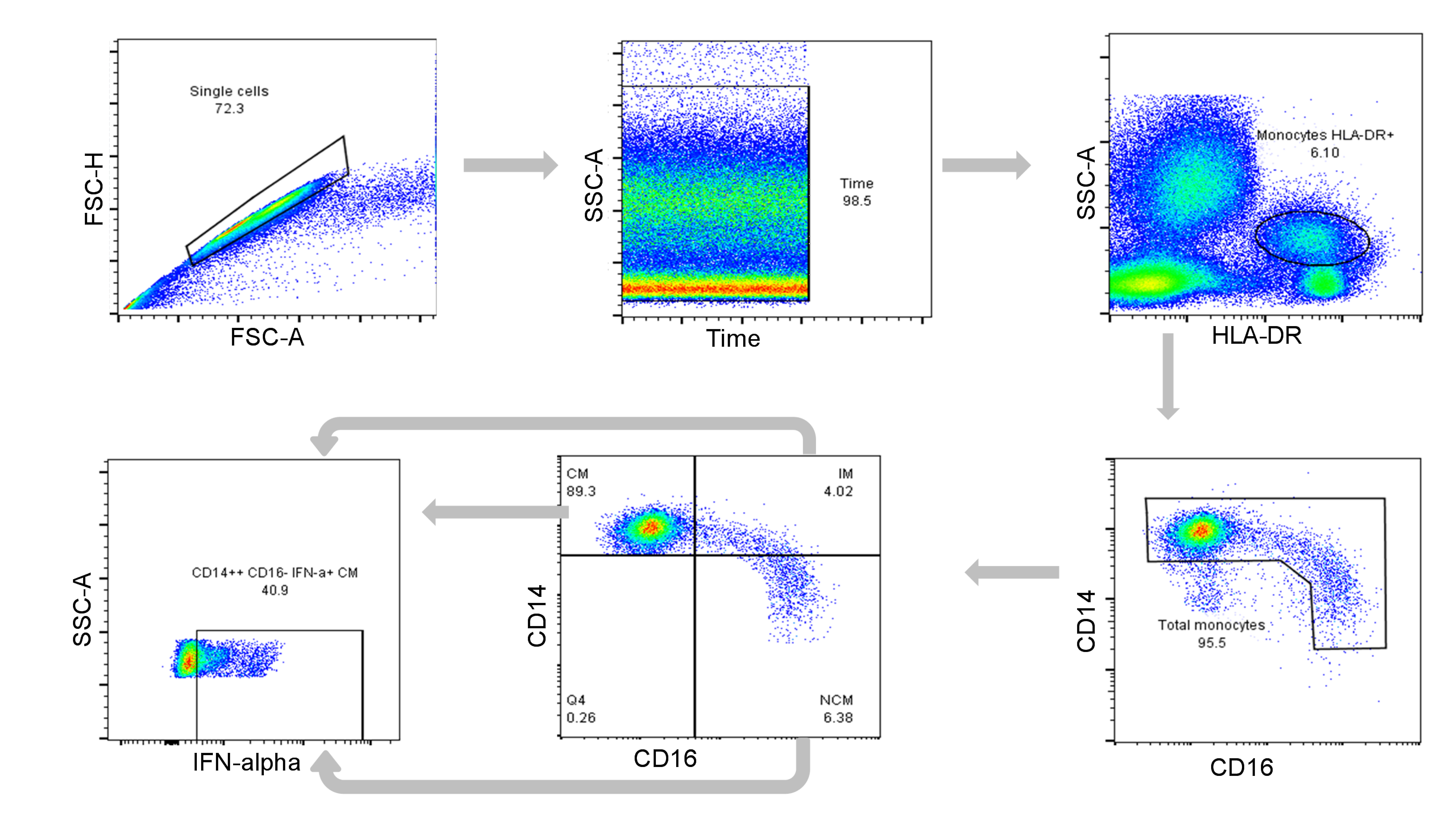

Supplement: Supplementary Figure 3 — Gating strategy for monocyte subsets. Gating strategy for selecting primary human HLA-DR+ monocyte subsets and measuring IFN-alpha precisely. FSC-H, forward scatter height; FSC-A, forward scatter area; SSC-A, side scatter area; HLA-DR, human leukocyte antigen-DR isotype; IFN-alpha. [file Image_3.tif]

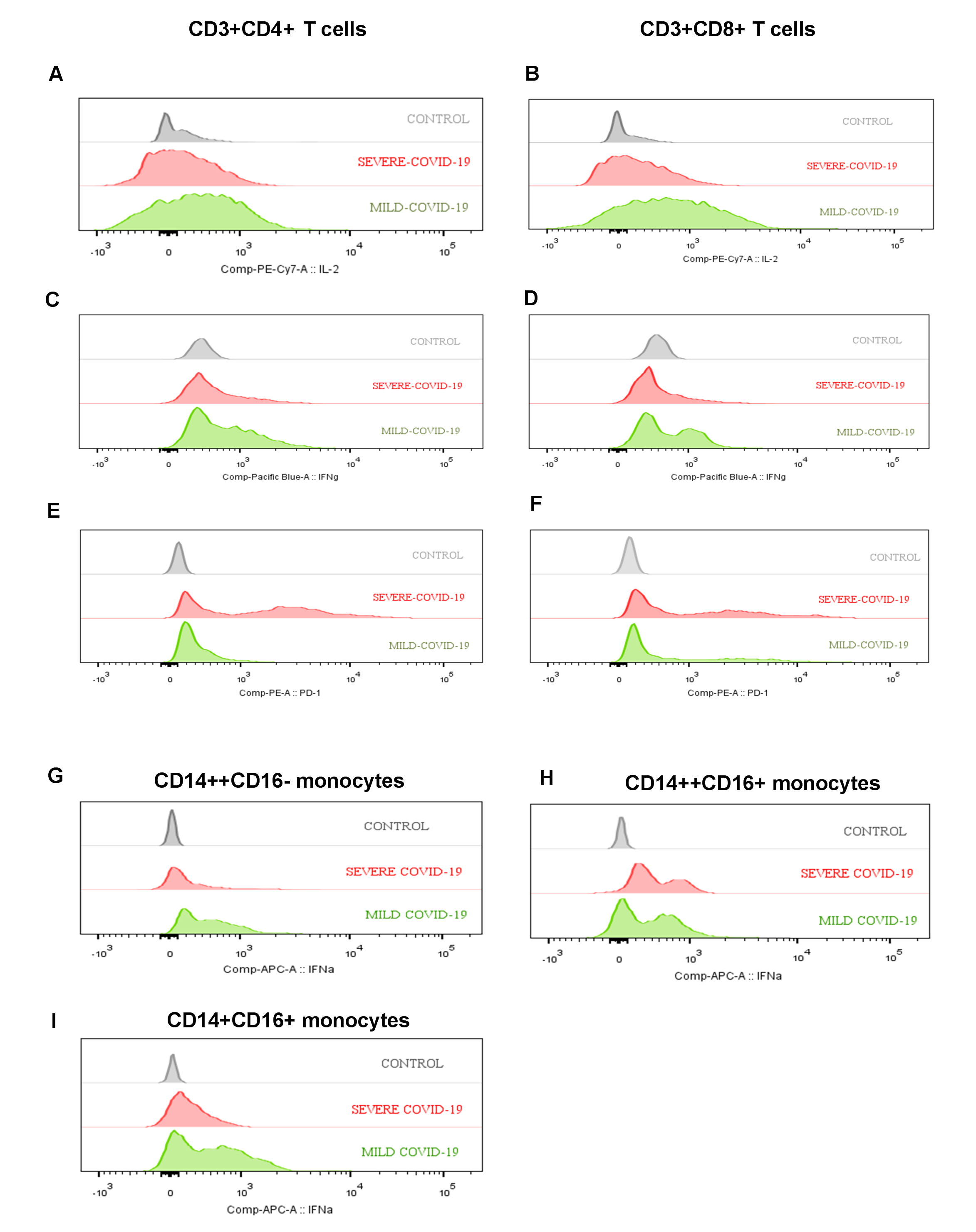

Supplement: Supplementary Figure 4 — Representative spectra of mean fluorescence intensity for IL-2, IFN-gamma, PD-1, and IFN-alpha in T cells and monocyte subsets. (A) Representative histograms show the comparison of CD3+CD4+ T cells expressing IL-2 in blood samples treated with polyclonal stimuli from participants who developed mild or severe COVID-19 during the follow-up. (B) Representative histograms show the comparison of CD3+CD8+ T cells expressing IL-2 in blood samples treated with polyclonal stimuli from participants who developed mild or severe COVID-19 throughout the follow-up. (C) Representative histograms show the comparison of CD3+CD4+ T cells expressing IFN-gamma in blood samples treated with polyclonal stimuli from participants who developed mild or severe COVID-19 during the follow-up. (D) Representative histograms show the comparison of CD3+CD8+ T cells expressing IFN-gamma in blood samples treated with polyclonal stimuli from participants who developed mild or severe COVID-19 throughout the follow-up. (E) Representative histograms show the comparison of CD3+CD4+ T cells expressing PD-1 in blood samples treated with polyclonal stimuli from participants who developed mild or severe COVID-19 during the follow-up. (F) Representative histograms show the comparison of CD3+CD8+ T cells expressing PD-1 in blood samples treated with polyclonal stimuli from participants who developed mild or severe COVID-19 throughout the follow-up. (G) Representative histograms show the comparison of CD14++CD16- classical monocytes expressing IFN-alpha in blood samples treated with polyclonal stimuli from participants who developed mild or severe COVID-19 throughout the follow-up. (H) Representative histograms show the comparison of CD14++CD16+ intermediate monocytes expressing IFN-alpha in blood samples treated with polyclonal stimuli from participants who developed mild or severe COVID-19 during the follow-up. (I) Representative histograms show the comparison of CD14+CD16+ non-classical monocytes expressing [file Image_4.tif]

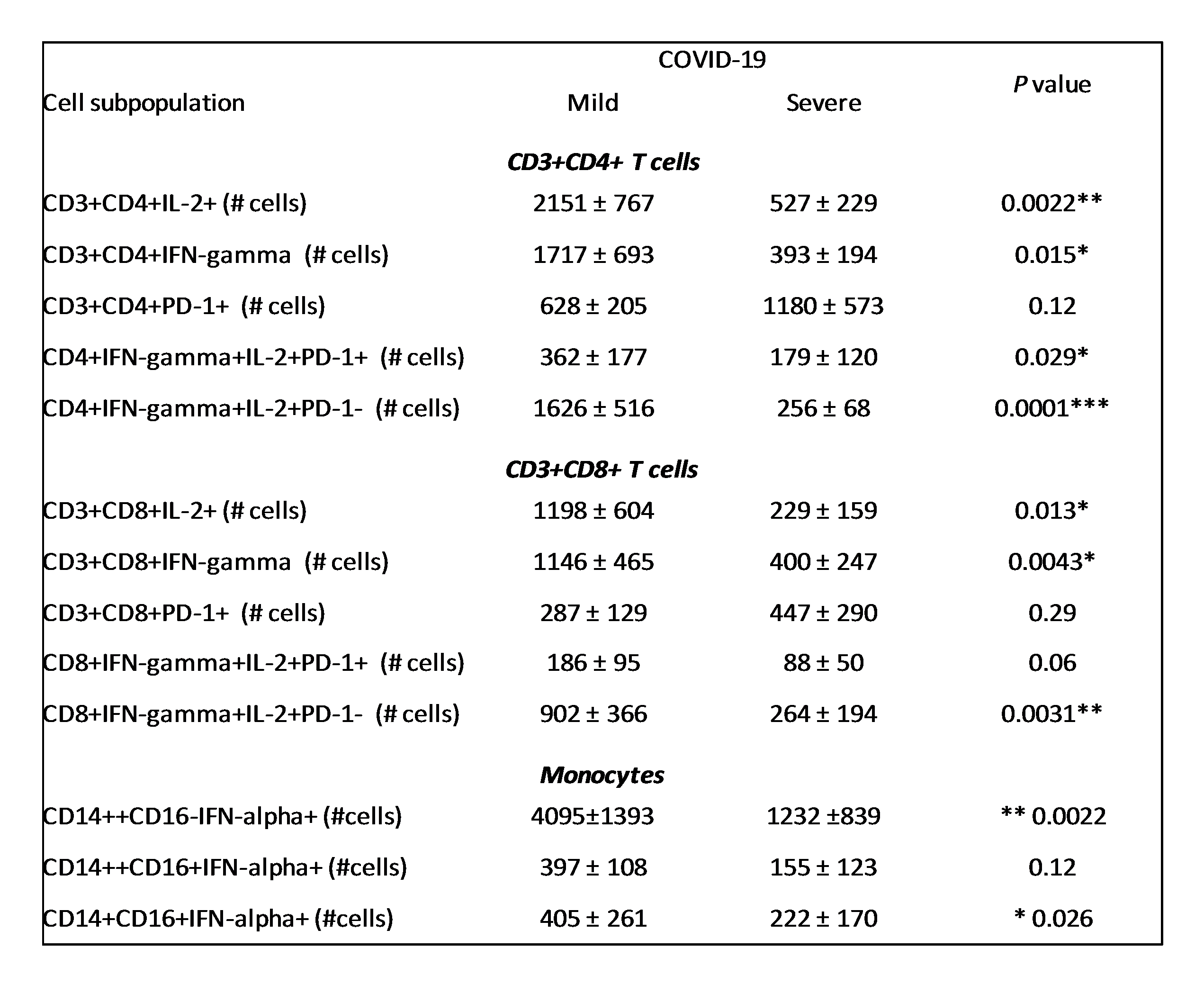

Supplement: Supplementary Figure 5 — Absolute cell numbers for percentages of T cells and monocyte subsets. We show absolute cell numbers for percentages of CD3+CD4+ T cells expressing IL-2, IFN-gamma, and PD-1 on top. We show absolute cell numbers for percentages of CD3+CD8+ T cells expressing IL-2, IFN-gamma, and PD-1 in the middle. We show absolute cell numbers for percentages of classical, intermediate, and non-classical monocytes expressing IFN-alpha on the bottom. We defined classical monocytes as CD14++CD16-, intermediate monocytes as CD14++CD16+, and non-classical monocytes as CD14+CD16+. We expressed data as mean ± standard deviation. We compared data using the unpaired Student’s T-test and considered differences significant when P < 0.05. IL-2, interleukin-2; IFN-gamma, interferon-gamma; PD-1, programmed cell death protein 1; IFN-alpha, interferon-alpha; COVID-19, coronavirus disease 2019. [file Image_5.tif]
